# Patient and Tumour Characteristics of Adult Head and Neck Soft Tissue Sarcomas: A Systematic Review and Meta-Analysis

**DOI:** 10.1155/2019/9725637

**Published:** 2019-05-22

**Authors:** Sakshi Andersen, Henriette Mann, Anders Krarup-Hansen, Christel Bræmer Lajer, Christian Grønhøj

**Affiliations:** ^1^Department of Otorhinolaryngology, Head and Neck Surgery and Audiology, Rigshospitalet, University of Copenhagen, Blegdamsvej 9, 2100 Copenhagen, Denmark; ^2^Department of Oncology, Herlev Hospital, Herlev Ringvej 75, 2730 Herlev, Denmark

## Abstract

**Background:**

Head and neck soft tissue sarcomas (HNSTS) constitute a rare and heterogeneous cancer entity. Management remains a challenge due to the rarity and varied biological behaviour among various subtypes. This systematic review examines the characteristics of tumours and patients with HNSTS.

**Materials and Methods:**

A systematic literature review and meta-analysis were performed using the electronic databases PubMed and Embase. Eight eligible studies were identified, and 13 variables were extracted from each study including 5-year overall survival (OS) rate and 5-year disease-free survival (DFS) rate.

**Results:**

We identified eight studies (*n* = 1,120 patients; 739 males (66%)) from six different countries). In total, 24 histological subtypes were found, and 20% of the sarcomas (*n* = 215) could not be subclassified. 607 sarcomas (57%) were <5 cm in diameter, and 945 sarcomas (84%) were grade 3. 1,059 patients (90%) underwent surgery. Estimated 5-year OS was 74% (95% CI; 0.63–0.84%) and 5-year DFS was 56% (95% CI; 38–74%).

**Conclusion:**

HNSTS holds a relative poor prognosis possibly explained by the heterogeneity of the disease. Treatment of HNSTS has shown to be highly diverse, underlining the importance of uniformed treatment guidelines in order to achieve improved survival outcomes.

## 1. Introduction

Head and neck soft tissue sarcomas (HNSTS) are a rare and heterogeneous group of malignancies accounting for approximately 1% of all head and neck malignancies. HNSTS represents 10% of all soft tissue sarcomas [1]. More than 80 histological subtypes are distinguished in the 2013 WHO classification [2]. Because of this rarity and the diverse clinical behaviours, management of HNSTS can be challenging and should be carried out in a multidisciplinary centre with expertise and experience in sarcomas [3].

Surgery remains to be the primary treatment choice, even though it is difficult to achieve free surgical margins due to the anatomy of the head and neck region [4]. When combined with surgery, radiotherapy may improve overall prognosis for some HNSTS depending on the histological type, whereas a better outcome is achieved for some histological subtypes when surgery is combined with chemotherapy, e.g., angiosarcoma, rhabdomyosarcoma, and synovial sarcoma [3, 5, 6].

The purpose of this study was to systematically review the literature on adult patients diagnosed with HNSTS and report the distribution of histological subtypes, treatment, and overall survival (OS) rates.

## 2. Materials and Methods

### 2.1. Search Strategy and Eligibility Criteria

The electronic databases PubMed and Embase were used for searching. The search strategy in PubMed included the following keywords: “Head and Neck,” “Oral Cavity,” “Pharynx,” and “Sarcoma.” The search strategy in Embase included the keywords “Head and Neck Sarcomas.” Studies published from 2000 until March 2018 reporting patient databases of soft tissue sarcomas in the head and neck region with a minimum of 20 patients (>18 years of age at diagnosis) were included. Studies reporting both localized disease and metastatic disease were included. Exclusion criteria were radiation-induced sarcomas, bone sarcomas, and studies that solely reported specific histological subtypes. Due to an update in WHO classification of soft tissue tumours in 1994, studies reporting patient cases from before 1994 were excluded.

### 2.2. Data Extraction

We extracted data on country, number of cases, period, gender, age, histological subtypes, tumour size, and grading according to the French Federation of Cancer Centres Sarcoma Group (grade I, grade II, grade III, and unknown). Furthermore, *T* stage (0, 1, 2, 3, and 4), treatment (surgery, radiotherapy, and/or chemotherapy), surgical margins (R0, R1, R2, and unspecified/unknown), median follow up, 5-year OS, and 5-year disease-free survival (DFS) were also extracted. We applied the model of random effects to perform a meta-analysis of 5-year OS and 5-year DFS. Data analysis was performed in Stata, and a *p* value < 0.05 was considered statistically significant.

## 3. Results

**Figure 1:**
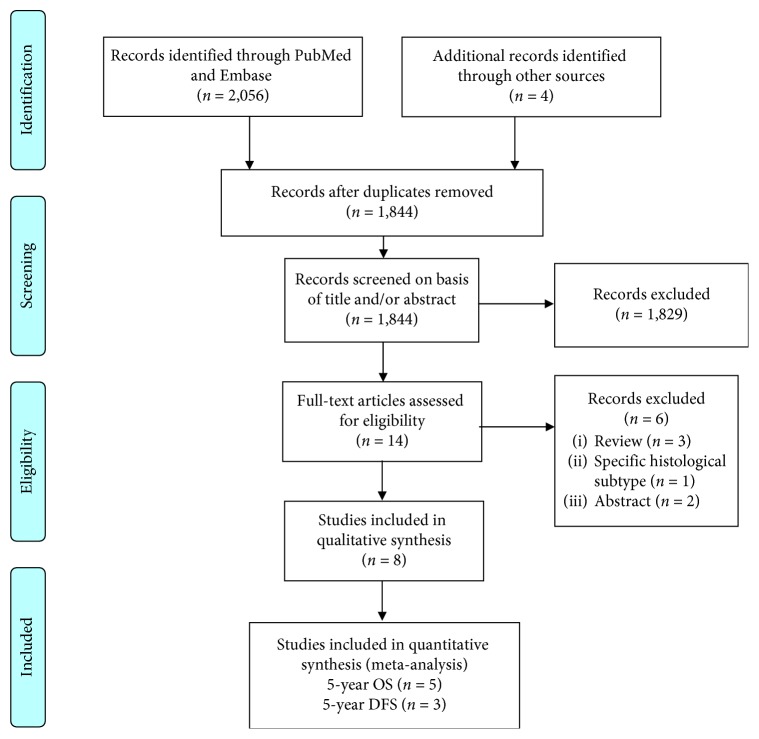
PRISMA 2009 flow diagram.

We identified 2,056 publications, of which eight studies (1,120 cases; 66% males) from six different countries met the inclusion criteria. All studies reported the median age, which ranged from 35 to 68.5 years. In four studies (*n* = 897), 73% of the patients were more than 50 years of age (Table 1 and Figure 1).

**Figure 2:**
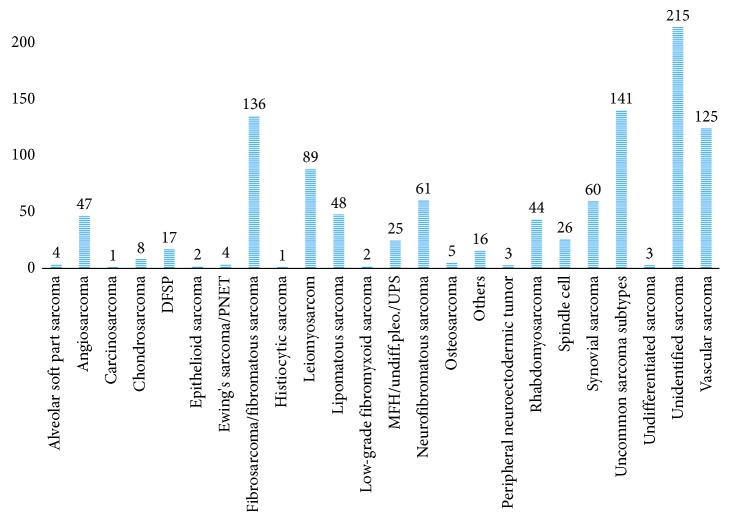
Histological subtypes.

Sarcomas with unidentified histology (*n* = 215) and uncommon sarcoma subtypes (*n* = 141) constituted one-third of the cohort (*n* = 1,083) being the most frequent histological subtypes. These were followed by fibrosarcomas/fibromatous sarcomas (*n* = 136), vascular sarcomas (*n* = 125), and leiomyosarcomas (*n* = 89). Noteworthy, in some of the included studies, the group “uncommon sarcomas” consisted of several sarcoma subtypes traditionally perceived as common sarcomas (e.g., rhabdomyosarcomas) (Table 2 and Figure 2). This was mainly seen in studies including a small number of cases. Hence, several of the cases classified as “uncommon sarcomas” could potentially be reclassified within one of the specified subtypes.

**Figure 3:**
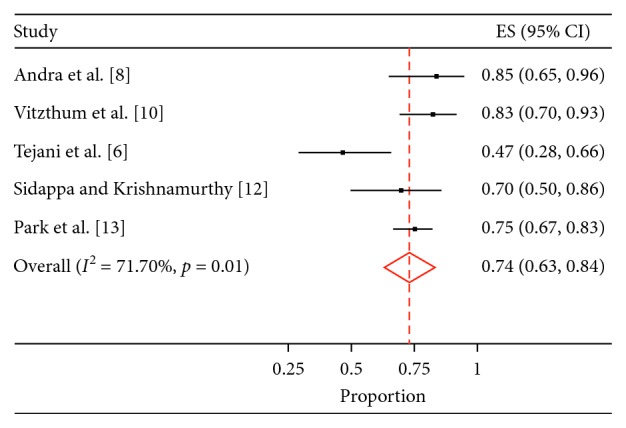
5-year overall survival.

**Figure 4:**
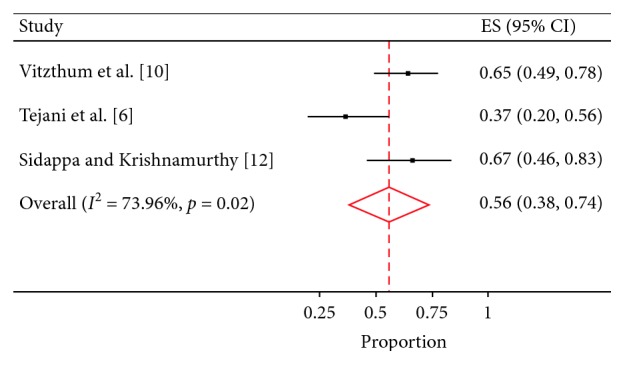
5-year disease-free survival.

In seven studies (*n* = 1,090), the median follow-up period ranged from 23.8 to 72 months. Five-year OS was reported in five studies, 5-year DFS was reported in three studies, and their values were 74% (95% CI; 0.63–0.84%) and 56 % (95% CI; 38–74%), respectively (Table 1 and Figures 3 and 4).

Six studies (*n* = 1,060) reported tumour size as a binary outcome (<5 cm or >5 cm), two studies reported median tumour size, and one study reported both mean tumour size and tumour size as a binary outcome. In 57% of the cases, the tumour diameter was <5 cm in diameter at the time of diagnosis. Mean tumour size ranged from 2.7 to 4.8 cm (*n* = 55). Tumour grading was stated in all studies included (*n* = 1,120). 84% of the tumours were grade III, 7% were grade II, 5% were grade I, and 3% were unspecified or unknown grade. The surgical resection margins were stated in seven studies (*n* = 1,092). 60% of the tumours had clear surgical margins (R0), 13% had microscopic involved margins (R1), 18% had macroscopic involved margins (R2), and 1% had unknown margins (Table 1).

All studies (*n* = 1,120) reported treatment strategy (Table 3).

## 4. Discussion

To our knowledge, this is the first systematic review and meta-analysis to explore patient and tumour characteristics including OS, DFS, and treatment strategies for patients with a HNSTS. In the meta-analysis, the pooled results showed a 5-year OS of approximately 75% and a 5-year DFS of nearly 50%.

Surgery remains the cornerstone of treatment of HNSTS, but the close relation to vital anatomic structures complicates this treatment strategy, explaining the relatively low 5-year DFS. Several factors influence the degree of surgical resection, e.g., tumour location and size, the extend of invasion, and the performance status of the patient [14]. Even though surgery with wide resection margins remains a difficult task, this review shows that 90% of the patients were treated with surgery (± radiation therapy and/or chemotherapy), and wide surgical margins were obtained in 60% of the cases.

In the present study, 53% of the patients were treated with radiation therapy with or without surgery. Studies in patients with truncal or extremity soft tissue sarcomas have demonstrated an improved local control following adjuvant radiation therapy in patients with large grade II and grade III sarcomas. Furthermore, retrospective studies have shown an approximately 10% improvement in OS following adjuvant radiation therapy in patients with grade III sarcomas [15]. Similarly, radiation therapy has also been found to improve OS in HNSTS [7].

In order to improve 5-year DFS and 5-year OS, the value of neoadjuvant chemotherapy in high-graded truncal or extremity soft tissue sarcomas has been investigated. However, the study was closed prematurely because superiority for neoadjuvant treatment was not to be expected [16]. Nevertheless, neoadjuvant chemotherapy still holds a potential improvement in the prognosis of HNSTS, and prospective, multicentre studies are needed in order to gain information about the effect of neoadjuvant treatment including chemotherapy and radiation therapy. Furthermore, in a study by Blay et al., early evaluation by a multidisciplinary team demonstrated a significant improvement in relapse-free survival [17]. Hence, HNSTS should only be managed by a specialist multidisciplinary environment in order to ensure a uniform treatment protocol [18].

More than 80 histological subtypes of HNSTS have been identified [2]. In order to predict risk of metastasis and relapse, it is important to identify the exact histological classification. In this systematic review, 215 sarcomas could not be classified, and additional 141 sarcomas were classified as “uncommon sarcomas.” It is remarkable that one-third of the cohort constituted of sarcomas with unknown histology and uncommon sarcoma subtypes. Noteworthy, the classification of soft tissue sarcomas has evolved considerably during the past decades, especially due to evolvement in immunohistochemical and genetic/molecular methods [19]. It is likely that a large number of the cases included in the present review would be reclassified if classified today. Furthermore, many of the cases where a specific pathological classification could not be obtained could probably be classified using modern methods. This may complicate applying the results of the present review on newly diagnosed patients with HNSTS. A high expertise in histological classification is crucial if more knowledge about behaviour and treatment of sarcomas must be achieved. It emphasizes the need of histological reevaluation of a special trained pathologist in the highest national level before treating and before publishing data of sarcomas.

The present systematic review and meta-analysis hold important limitations. Inclusion and exclusion criteria as well as staging and treatment varied between the included studies, which complicates interpretation. The included studies reported a few cases of sarcomas, which were not classified as soft tissue sarcomas; however, these constituted only a small part (1.3%) of the total cases included. The included studies originated from six countries, and the majority of the cases were obtained from a single study. All studies were retrospective, and the cases were obtained during long follow-up periods.

## 5. Conclusions

HNSTS is a rare and heterogeneous tumour group with great differences in OS and DFS. The pooled results showed a 5-year OS of approximately 75% and a 5-year DFS of nearly 50%. However, studies show varying prognoses demonstrating the difficulty in treating HNSTS underlining the importance of uniformed treatment guidelines in order to achieve improved survival outcomes.

## Figures and Tables

**Table 1:** Patient data and characteristics.

	Mahmoud et al. [7]		Andrä et al. [8]		González-González et al. [9]		Vitzthum et al. [10]		Tejani et al. [6]		Penel et al. [11]		Sidappa and Krishnamurthy [12]		Park et al. [13]		Total	
Country	USA		Germany		Mexico		USA		USA		France		India		Korea		*N*	%
Years	2004–2013		2003–2012		2004–2009		1998–2012		1999–2009		1997–2002		1996–2005		1995–2012			
	*N*	%	*N*	%	*N*	%	*N*	%	*N*	%	*N*	%	*N*	%	*N*	%		
Cases	788		26		51		48		30		28		27		122		1120	
Male	539	68	18	69	31	61	34	71	20	67	15	54	17	63	65	53	739	66
Median age	63		64		43.1		68.5		50		45.7		35		46		—	
<50	181	23^a^	—		34	67	—		12	40	11	39	—		—		238	27
>50	607	77	—		17	33^b^	—		18	60	17	61^b^	—		—		659	73
Tumour size																		
<5 cm	443	56	—		15	29^c^	33	69^c^	13	43	—		17	63	86	70^c^	607	57
>5 cm	345	44^d^	—		36	71	15	31	11	37	—		10	37	36	30	453	43
Mean tumour size (cm)	4.5^e^		4.6^e^		—		—		—		2.7		4.8				—	
Grade (FNCLCC)																		
High/G3	788	100	17	65	30	59	28	58^f^	23	77	16	57	21	78^g^	22	18	945	84
Intermediate/G2	0		8	31	10	20	0		3	10	8	29	0		53	43	82	7
Low/G1	0		1	4	11	22	6	13	2	7	2	7	6	22	30	25	58	5
Unspecified/unknown	0		0		0		14	29	2	7	2	7	0		17	14	35	3
*T* stage																		
0	—		—		—		—		—		—		—		—		—	
1	—		14	54	13	25	—		12	40	—		—		—		39	23
2	—		12	46	38	75	—		18	60	—		—		—		68	40
3	—		—		—		—		—		—		—		—		—	
4	—		—		—		—		—		—		—		—		—	
Surgical margins^†^																		
R0	497	63	10	39	35	69	41	85	17	57	—		20	74	76	62	696	60
R1	111	14	6	23	4	8	—		7	23	—		6	22^h^	20	16	154	13
R2	180	23	5	19	13	25	3	6	—		—		—		7	6	208	18
Unspecified/unknown	—		5	19	—		4	8	—		—		—		—		9	1
5-year OS	—			82	—			83		46	—		68		75		—	
5-year DFS	—		—		—			63		35	—		65		—		—	
Median follow-up (months)	45		39		23.8		57.6		—		27		59		72		—	

^a^≤50, ^b^≥50, ^c^≤5, ^d^≥5, ^e^median, ^f^grade 2 + 3, ^g^grading system not specified in the article, and ^h^R1 + R2. ^†^R0 = negative surgical margins/clear margins, R1 = microscopic positive surgical margins/microscopic involved margins, and R2 = macroscopic positive margins/macroscopic involved margins.

**Table 2:** Histological subtypes.

	Mahmoud et al. [7]	Andrä et al. [8]	González-González et al. [9]^*∗*^	Vitzthum et al. [10]^*∗*^	Tejani et al.[6]	Penel et al. [11]^*∗*^	Sidappa and Krishnamurthy [12]	Park et al. [13]	Total
Alveolar soft part sarcoma	—	—	—	—	1	—	—	3	4
Angiosarcoma	—	9	—	14	4	5	—	15	47
Carcinosarcoma	—	—	—	—	1	—	—	—	1
Chondrosarcoma	—	—	—	—	—	—	—	8	8
DFSP^▪^	—	—	—	1	—	—		16	17
Epithelioid sarcoma	—	—	—	—	1	1	—	—	2
Ewing's sarcoma/PNET^◊^	—	—	—	—	—	—	—	4	4
Fibrosarcoma/fibromatous sarcoma^○^	125	—	—	—	1	2	1	7	136
Histiocytic sarcoma	—	—	—	—	1	—	—	—	1
Leiomyosarcoma	76	—	—	5	3	—	1	4	89
Lipomatous sarcoma^∆^	31	—	—	3	4	—	—	10	48
Low-grade fibromyxoid sarcoma	—	—	—	—	—	—	—	2	2
MFH/undiff. pleo./UPS^∞^	—	5	—	9	—	2	6	3	25
Neurofibromatous sarcoma^□^	46	—	9	1	—	—	5	—	61
Osteosarcoma	—	—	—	—	—	—	—	5	5
Others^•^	—	8	—	3	—	—	—	5	16
Peripheral neuroectodermic tumour	—	—	—	—	—	3	—	—	3
Rhabdomyosarcoma	—	—	7	1	5	7	—	24	44
Spindle cell	—	—	—	4	3	—	10	9	26
Synovial sarcoma	34	4	—	6	6	2	1	7	60
Uncommon sarcoma subtypes^†^	139	—	—	—	—	—	2	—	141
Undifferentiated sarcoma	—	—	—	—	—	3	—	—	3
Unidentified sarcoma^♦^	215	—	—	—	—	—	—	—	215
Vascular sarcoma^‡^	122	—	—	2	—	—	1	—	125

^*∗*^Not all of the histological subtypes were reported. ^▪^Dermatofibrosarcoma protuberans. ^◊^Primitive neuroectodermal tumour. ^○^Including fibrosarcoma NOS, fibromyxoma, and fibrous histiocytoma. ^∆^Including myxosarcoma, angiomyxoma, atypical lipoma, liposarcoma NOS, fibromyxolipoma, myxoid, round cell, pleomorphic, mixed, and dedifferentiated liposarcoma, and spindle cell lipoma. ^∞^MFH = malignant fibrous histiocytoma, undiff. pleo. = undifferentiated pleomorphic sarcoma. UPS = unclassified pleomorphic sarcoma. ^□^Including neurosarcoma and malignant peripheral nerve sheath tumour. ^•^Andrä et al. [8], not otherwise specified; Vitzthum et al. [10], dendritic cell sarcoma, chordoma, and hemangioengothelioma; Park et al. [13], folicular dendritic cell sarcoma and teratocarcinosarcoma. ^†^Including clear cell, dermatofibrosarcoma, rhabdomyosarcoma, epithelioid, desmoplastic small round cell tumour, fascial, infantile, angiomatoid, fibrous histiocytoma, rhabdoid, giant cell tumour of soft part, and alveolar soft part. ^♦^Sarcomatosis not otherwise specified (NOS), spindle cell, giant cell, small cell, and undifferentiated sarcoma. ^‡^Including hemangiosarcoma, malignant hemangioendothelioma, epithelioid hemangioendothelioma, malignant hemangiopericytoma, hemangiopericytoma, and lymphangiosarcoma.

**Table 3:** Treatment.

	Mahmoud et al. [7]		Andrä et al. [8]		González-González et al. [9]		Vitzthum et al. [10]		Tejani et al. [6]		Penel et al. [11]		Sidappa and Krishnamurthy [12]		Park et al. [13]		Total	
	*N*	%	*N*	%	*N*	%	*N*	%	*N*	%	*N*	%	*N*	%	*N*	%	*N*	%
Surgery	788	100	21	81	48	94	30	63	24	80	19	68	26	96	103	84	1059	90
Radiation therapy	414	53	26	26	33	65	34	71	18	60	14	50	21	78	65	53	625	53
Chemotherapy	126	16	13	13	12	24	2	4	10	33	8	29	0	—	42	34	213	18

## References

[1] O’Neill J. P., Bilsky M. H., Kraus D. (2013). Head and neck sarcomas: epidemiology, pathology, and management. *Neurosurgery Clinics of North America*.

[2] Karanian M., Coindre J.-M. (2015). Quatrième édition de la classification OMS des tumeurs des tissus mous. *Annales de Pathologie*.

[3] Casali P. G., Abecassis N., Bauer S. (2018). Soft tissue and visceral sarcomas: ESMO-EURACAN clinical practice guidelines for diagnosis, treatment and follow-up. *Annals of Oncology*.

[4] Woods R. H., Potter J. A., Reid J. L. (2017). Patterns of head and neck sarcoma in Australia. *ANZ Journal of Surgery*.

[5] Tudor-Green B., Gomez R., Brennan P. A. (2017). Current update on the diagnosis and management of head and neck soft tissue sarcomas. *Journal of Oral Pathology & Medicine*.

[6] Tejani M., Galloway T., Lango M., Ridge J., Von Mehren M. (2013). Head and neck sarcomas: a comprehensive cancer center experience. *Cancers*.

[7] Mahmoud O., Beck R., Kalyoussef E. (2017). Adjuvant therapies utilization pattern and survival outcomes in high-grade head and neck soft tissue sarcoma; a population based study. *Oral Oncology*.

[8] Andrä C., Rauch J., Li M. (2015). Excellent local control and survival after postoperative or definitive radiation therapy for sarcomas of the head and neck. *Radiation Oncology*.

[9] González-González R., Bologna-Molina R., Molina-Frechero N., Domínguez-Malagon H. R. (2012). Prognostic factors and treatment strategies for adult head and neck soft tissue sarcoma. *International Journal of Oral and Maxillofacial Surgery*.

[10] Vitzthum L. K., Brown L. B., Rooney J. W., Foote R. L. (2016). Head and neck soft tissue sarcomas treated with radiation therapy. *Rare Tumors*.

[11] Penel N., Van Haverbeke C., Lartigau E. (2004). Head and neck soft tissue sarcomas of adult: prognostic value of surgery in multimodal therapeutic approach. *Oral Oncology*.

[12] Sidappa K. T., Krishnamurthy A. (2011). Adult soft-tissue sarcomas of the head and neck. *Indian Journal of Cancer*.

[13] Park J. T., Roh J. L., Kim S. O. (2015). Prognostic factors and oncological outcomes of 122 head and neck soft tissue sarcoma patients treated at a single institution. *Annals of Surgical Oncology*.

[14] De Bree R., Van Der Waal I., De Bree E., René Leemans C. (2010). Management of adult soft tissue sarcomas of the head and neck. *Oral Oncology*.

[15] Larrier N. A., Czito B. G., Kirsch D. G. (2016). Radiation therapy for soft tissue sarcoma: indications and controversies for neoadjuvant therapy, adjuvant therapy, intraoperative radiation therapy, and brachytherapy. *Surgical Oncology Clinics of North America*.

[16] Gronchi A., Ferrari S., Quagliuolo V. (2017). Histotype-tailored neoadjuvant chemotherapy versus standard chemotherapy in patients with high-risk soft-tissue sarcomas (ISG-STS 1001): an international, open-label, randomised, controlled, phase 3, multicentre trial. *Lancet Oncology*.

[17] Blay J.-Y., Soibinet P., Penel N. (2017). Improved survival using specialized multidisciplinary board in sarcoma patients. *Annals of Oncology*.

[18] Blay J. Y. (2018). Management of sarcoma patients: centralization in reference centers to fragmentation of systemic treatment. *Current Opinion in Oncology*.

[19] Jo V. Y., Doyle L. A. (2016). Refinements in sarcoma classification in the current 2013 world health organization classification of tumours of soft tissue and bone. *Surgical Oncology Clinics of North America*.

